# Zac1 plays a key role in the development of specific neuronal subsets in the mouse cerebellum

**DOI:** 10.1186/1749-8104-6-25

**Published:** 2011-05-18

**Authors:** Seung-Hyuk Chung, Hassan Marzban, Kimberly Aldinger, Rajiv Dixit, Kathleen Millen, Carol Schuurmans, Richard Hawkes

**Affiliations:** 1 Department of Cell Biology and Anatomy, Hotchkiss Brain Institute, and Genes and Development Research Group, Faculty of Medicine, University of Calgary, Calgary, Alberta T2N 4N1, Canada; 2 Department of Human Genetics, University of Chicago, Chicago, Illinois 60637, USA; 3 Hotchkiss Brain Institute, and Genes and Development Research Group, Faculty of Medicine, University of Calgary, Calgary, Alberta T2N 4N1, Canada

## Abstract

**Background:**

The cerebellum is composed of a diverse array of neuronal subtypes. Here we have used a candidate approach to identify *Zac1*, a tumor suppressor gene encoding a zinc finger transcription factor, as a new player in the transcriptional network required for the development of a specific subset of cerebellar nuclei and a population of Golgi cells in the cerebellar cortex.

**Results:**

We found that Zac1 has a complex expression profile in the developing cerebellum, including in two proliferating progenitor populations; the cerebellar ventricular zone and the external granular layer overlying posterior cerebellar lobules IX and X. Zac1 is also expressed in some postmitotic cerebellar neurons, including a subset of GABAergic interneurons in the medial cerebellar nuclei. Notably, GABAergic interneurons in the cerebellar nuclei are derived from the cerebellar ventricular zone, where Zac1 is also expressed, consistent with a lineage relationship between these two Zac1^+ ^populations. Zac1 is also expressed in a small subset of cells in the posterior vermis, including some neurogranin-immunoreactive (NG^+^) Golgi cells, which, based on short-term birthdating, are derived from the EGL, where Zac1 is also expressed. However, Zac1^+ ^cells and NG^+ ^Golgi cells in the cerebellar cortex also display unique properties, as they are generated within different, albeit overlapping, time windows. Finally, consistent with the expression profile of Zac1, two conspicuous abnormalities were found in the cerebellum of *Zac1 *null mice: the medial cerebellar nuclei, and not the others, were significantly reduced in size; and the number of Golgi cells in cerebellar lobule IX was reduced by approximately 60% compared to wild-type littermates.

**Conclusions:**

The data presented here indicate that the tumor suppressor gene *Zac1 *is expressed in a complex fashion in the developing cerebellum, including in two dividing progenitor populations and in specific subsets of postmitotic neurons, including Golgi cells and GABAergic neurons in the medial nuclei, which require Zac1 for their differentiation. We thus conclude that Zac1 is a critical regulator of normal cerebellar development, adding a new transcriptional regulator to the growing list of factors involved in generating neuronal diversity in the developing cerebellum.

## Background

The cerebellum has two major cellular divisions - the cerebellar cortex and the cerebellar nuclei. The cerebellar cortex is subdivided into distinct laminae, each with characteristic cellular compositions, and the cortical sheet is highly parcellated into a stereotyped array of transverse zones and parasagittal stripes {for example, [1]). The efferent output of the cerebellar cortex terminates on the cerebellar and vestibular nuclei, which also possess a complex cytoarchitecture [2]. Two progenitor cell zones are thought to give rise to all cerebellar neurons: the cerebellar ventricular zone (VZ) and the rhombic lip.

Transcriptional regulators control neuronal fate specification and differentiation events throughout the developing central and peripheral nervous systems. Notably, several basic-helix-loop-helix factors, including Ptf1a and Atoh1/Math1 in the cerebellum [3] and Neurog2 and Ascl1/Mash1 in the telencephalon [4,5], function in opposition to control binary fate choices between glutamatergic and GABAergic neurotransmitter identities. However, in the cerebellum, neuronal classification based solely on neurotransmitter phenotypes does not accurately portray the wide diversity of neuronal subtypes. Consequently, factors other than Ptf1a and Atoh1 must contribute to the specification and differentiation of distinct cerebellar neurons. We focused our analyses on *Zac1 *(*zinc finger protein that regulates apoptosis and cell-cycle arrest*; also known as *Lot1 *(*lost on transformation*) and *Plagl1 *(*pleiomorphic adenoma gene like 1*)), which encodes a seven C2H2 zinc finger domain protein that can act as an activator, repressor, co-activator or co-repressor of transcription [6-10]. *Zac1 *is a maternally imprinted gene, as are several genes involved in growth control and for which gene dosage is critical [11,12]. Accordingly, both the overexpression of *Zac1 *in humans (due to loss of imprinting) and a null mutation in mouse result in intrauterine growth restriction [13-15]. *Zac1 *is also a tumor suppressor gene, promoting cell cycle arrest and apoptosis in cell lines, while germline mutations are associated with numerous carcinomas in humans [16-20]. During mouse embryonic and early postnatal development, *Zac1 *is highly expressed in numerous neuroepithelia with active proliferation, including the neuroepithelium of the fourth ventricle and the external granular layer (EGL) of the cerebellum [21,22]. Recently, *Zac1 *was shown to elicit apoptosis and cell cycle arrest in the developing murine retina [23] and to influence retinal cell fate decisions in both mouse [23] and *Xenopus *[24]. Specifically, in *Zac1 *null mutant mice, the retina becomes hypercellular in late retinogenesis with a specific expansion of amacrine cell and rod photoreceptor populations [23].

Here we show that Zac1 is expressed in a restricted manner in the developing cerebellum, suggestive of a role in the formation of two specific subsets of GABAergic neurons - a specific sub-group of the medial cerebellar nuclei, and a subset of Golgi cells. These hypotheses were confirmed by analysis of the cerebellum of *Zac1 *null mice: specific defects were identified in both the medial nuclei and Golgi cells of the posterior vermis.

## Materials and methods

### Animal maintenance and tissue processing

All animal procedures conformed to institutional regulations and the Guide of the Care and Use of Experimental Animals from the Canadian Council of Animal Care. CD1 and C57BL/6 mice were obtained from Charles River Laboratories (St Constant, PQ, Canada) and maintained in the Animal Resource Centre at the University of Calgary. The *Zac1 *mutant allele [15] was maintained on a C57BL/6 background and genotyping was performed as described [23]. *Zac1*^+m/- ^heterozygous embryos were effectively *Zac1 *null mutants due to maternal imprinting of the *Zac1 *locus [25], and were generated by crossing *Zac1*^*+/- *^heterozygous males to C57BL/6 females. *Atoh1*^*lacZ *^mice [26], a generous gift of Dr H Zoghbi, were maintained as heterozygous breeding pairs and genotyped as described. Embryos were staged using embryonic day (E) 0.5 as the day of the vaginal plug. For postnatal and adult analyses, mice were deeply anaesthetized with sodium pentobarbital (100 mg/kg, intraperitoneally) and transcardially perfused with 0.9% NaCl in 0.1 M phosphate buffer (pH 7.4) followed by fixation in 4% paraformaldehyde with 0.02% glutaraldehyde in 0.1 M phosphate buffer (pH 7.4). The brains were then removed, post-fixed in 4% paraformaldehyde at 4°C for 24 hours and stored in Millonig's solution (pH 7.6). Series of 40-μm transverse or sagittal sections were cut through the extent of the cerebellum on a cryostat.

### Immunohistochemistry

Immunohistochemistry was carried out on free-floating sections as described previously [27,28]. Briefly, tissue sections were washed thoroughly in phosphate-buffered saline (PBS), and blocked with 10% normal goat serum (Jackson Immunoresearch Laboratories, West Grove, PA, USA). Primary antibodies were diluted in PBS containing 0.1% Triton-X100 and 5% bovine serum albumin and sections incubated for 16 to 18 hours at room temperature. For immunoperoxidase labeling, sections were washed thoroughly in PBS and then incubated in biotinylated goat anti-rabbit or biotinylated goat anti-mouse Ig antibody (1:1,000 in blocking solution; Jackson Immunoresearch Laboratories) for 2 hours at room temperature. Antibody binding was revealed with a Vectastain ABC Staining Kit (Vector Laboratories Inc., Burlingame, CA, USA) according to the manufacturer's instructions. Sections were mounted on slides, dehydrated through an alcohol series, cleared in Histoclear, and cover-slipped with Entellan mounting medium (BDH Chemicals, Toronto, ON, Canada). For fluorescent immunolabeling, CY3-conjugated goat anti-rabbit secondary antibody (Alexa conjugated antibodies) and CY2-conjugated goat anti-mouse secondary antibodies were used as above (1:1,000; Jackson Immunoresearch Laboratories). After 2 hours the sections were washed in PBS, mounted onto chrome-alum and gelatin subbed slides, air-dried overnight, cleared, and cover-slipped with non-fluorescing mounting medium (Fluorsave Reagent, Calbiochem, La Jolla, CA, USA).

The following primary antibodies were used: rabbit anti-Zac1 (1:500) [7]; rabbit anti-neurogranin (anti-NG; 1:1,000; Chemicon, Temecula, CA, USA) [29]; mouse anti-calbindin (1:1,000; Swant Inc., Bellinzona, Switzerland); rabbit anti-T-box brain gene 1 (Tbr1; 1:1,000; AbCam Inc., Cambridge, MA, USA) [30]; mouse anti-zebrin II (1:200) [31]; mouse anti-5-bromo-2-deoxyuridine (anti-BrdU; 1:500; Developmental Studies Hybridoma Bank, Iowa City, IA, USA); guinea-pig anti-mouse metabotropic glutamate receptor (mGluR)1α antibody (1:500) [32]; rat anti-BrdU (1:500; Cedarlane Laboratories Limited, Hornby, ON, Canada); mouse anti-calretinin (1:1,000; Swant Inc.); rabbit anti-kinesin light chain 3 (anti-KLC3; 1:500) [33].

Photomicrographs were captured with a SPOT Cooled Color digital camera (Diagnostic Instruments Inc (Sterling Heights, MI, USA). running under and assembled in Adobe Photoshop. Confocal images were obtained using an Olympus FV300 confocal microscope. The images were cropped and corrected for brightness and contrast but not otherwise manipulated.

### Golgi cell birthdating

Golgi cells were birthdated by administering intraperitoneal injections of 6 mg BrdU (as a 10 mg/ml solution in sterile saline) to timed-pregnant dams (E11, E12, E13, E14, E15, E16, and E17). The dams were perfused at postnatal day (P) 5 or P20 and the cerebella from the pups immersion post-fixed and processed for anti-BrdU immunocytochemistry.

### Primary cerebellar cultures

Primary cerebellar cultures were prepared from E18 CD1 mice and maintained 21 days *in vitro *according to the methods of Furuya *et al. *[34] and Tabata *et al. *[35] with some modifications [27]. Briefly, the entire cerebellum was removed and kept in ice-cold Ca2^+^/Mg2^+^-free Hank's balanced salt solution (HBSS) containing gentamicin (10 μg/ml) and glucose (6 mM). The cerebella were incubated at 34°C for 12 minutes in HBSS containing 0.1% trypsin. After washing, the cerebella were gently triturated in HBSS containing DNase I (5 U/ml) and 12 mM MgSO_4 _until the cell mass was no longer visible. The cells were collected by centrifugation and resuspended in seeding medium (1:1 Dulbecco's modified Eagle's medium and F-12) supplemented with putrescine (100 μM), sodium selenite (30 nM), L-glutamine (1.4 mM), gentamicin (5 μg/ml), and 10% heat-inactivated fetal bovine serum. The cell suspensions were seeded on poly-L-ornithine coated glass coverslips (12 mm) at a density of 5 × 10^6 ^cells/ml, with each coverslip in a well of a 6 × 4 well dish. After 6 to 8 hours incubation in a CO_2 _incubator (100% humidity, 37°C, 5% CO_2_), 500 μl of culture medium further supplemented with transferrin (200 μg/ml), insulin (20 μg/ml), progesterone (40 nM), and tri-iodothyronine (0.5 ng/ml) was added to each culture well. Every 7 days, half of the medium in each dish was replaced with fresh culture medium additionally supplemented with cytosine arabinoside (4 μM) and bovine serum albumin (100 μg/ml).

### Cell counts

To estimate the number of Golgi cells in wild-type and null cerebellar cortices, NG-immunoreactive Golgi cells were counted from transverse sections through the full width of the vermis of cerebellar lobules V, VI and IX (+/+, N = 4; +^m^/-, N = 3).

## Results

### Zac1 and Tbr1 distinguish GABAergic and glutamatergic subdivisions of the medial cerebellar nuclei

Previous studies have used RNA *in situ *hybridization to characterize the distribution of *Zac1 *transcripts in the developing cerebellum, revealing a restricted cellular pattern suggestive of a role for this factor in the specification and/or differentiation of specific cerebellar cell populations [22]. To better understand how *Zac1 *might function during cerebellar development, we performed a detailed spatiotemporal expression analysis using Zac1-specific antisera [7]. We first examined whether Zac1 protein was detected in either of the cerebellar progenitor cell zones - the rhombic lip or the cerebellar VZ - from which all cerebellar neurons are derived. To provide a frame of reference, we compared the expression of Zac1 to Tbr1, a T-box transcription factor that is expressed in a subset of rhombic-lip derived, glutamatergic neurons.

**Figure 1:**
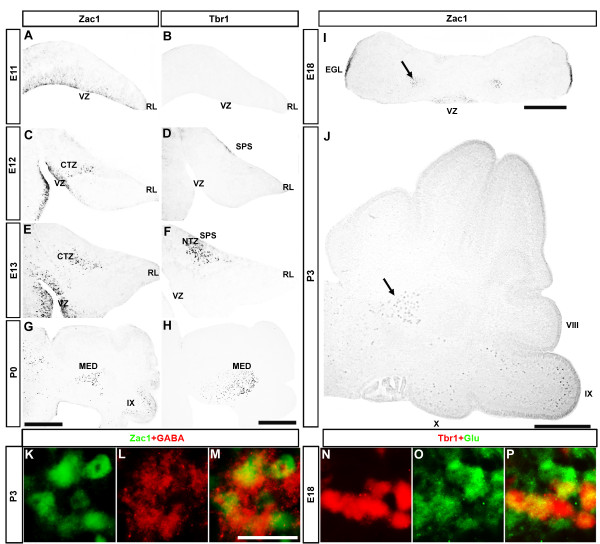
**Zac1 and Tbr1 mark distinct cell populations in the developing cerebellum**. **(A-H) **Expression of Zac1 (A,C,E,G) and Tbr1 (B,D,F,H) in E11 (A,B), E12 (C,D), E13 (E,F) and P0 (G,H) neighboring sagittal sections of the cerebellum. **(I,J) **Expression of Zac1 in E18 transverse sections (I) and P3 sagittal sections (J) of the cerebellum. Arrows in (I,J) mark the medial nucleus. **(K-M) **Transverse section of P3 cerebellum labeled with anti-Zac1 (green) and anti-GABA (red). **(N-P) **Transverse section of E18 cerebellum labeled with anti-Tbr1 (red) and anti-glutamate (green). Scale bars: 500 μm (H,I); 250 μm (J); 25 μm (M). CTZ, cortical transitory zone; MED, medial nucleus; NTZ, nuclear transitory zone; RL, rhombic lip; SPS, subpial stream; VZ, ventricular zone.

At E11, Zac1 immunoreactivity was detected in the cerebellar VZ overlying the fourth ventricle and not in the rhombic lip (Figure 1A). In contrast, Tbr1, which labels a subset of postmitotic cells derived from the rhombic lip, was not expressed at E11 (Figure 1B). By E12, Zac1^+ ^cells were detected in the VZ and in a continuous stream of cells that appeared to be migrating dorsally from the VZ towards the cortical transitory zone (Figure 1C). Conversely, at E12, Tbr1 labeled a subpial stream of cells at the dorsal surface of the cerebellar anlage, a cell population that is derived from rhombic lip progenitors [36]. At E13, Zac1^+ ^cells were evident in the VZ and in the cortical transitory zone, labeling a discrete cluster in the center of the cerebellar anlage (Figure 1E). The Zac1^+ ^cell clusters were immediately adjacent to more dorsal clusters of Tbr1^+ ^cells that had formed in the nuclear transitory zone after migrating through the subpial stream (Figure 1F) [36]. By P0, Zac1^+ ^cells formed symmetrical cell aggregations located ventral to the medial cerebellar nuclei (Figure 1G,I) and rostral to the Tbr1^+ ^subdivision of the medial nuclei (Figure 1H).

Zac1 and Tbr1 are thus expressed in distinct subdivisions of the medial cerebellar nuclei. Such a segregation could be explained if Zac1 was expressed in GABAergic cells derived from the cerebellar VZ, as suggested by its expression pattern, whereas Tbr1 labeled glutamatergic cells derived from the rhombic lip. Indeed, double-immunofluorescence labeling of transverse sections confirmed that Zac1^+ ^cells in the cerebellar nuclei are GABAergic (P3; Figure 1K-M) whereas Tbr1^+ ^cells are glutamatergic (E18.5; Figure 1N-P) [36]. We have thus identified Zac1 as a novel marker of GABAergic neurons in the medial nuclei of the cerebellum.

### Zac1 is expressed in the external granular layer and its derivatives

**Figure 2:**
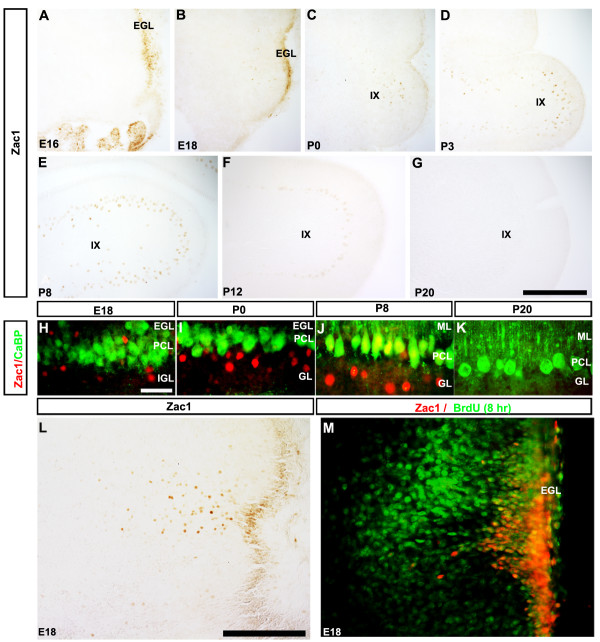
**Zac1 is expressed in subsets of Golgi cells and Purkinje cells in the cerebellar cortex**. **(A-G) **Expression of Zac1 in sagittal sections of the cerebellum at E16 (A), E18 (B), P0 (C), P3 (D), P8 (E), P12 (F) and P20 (G). Zac1^+ ^cells in the granular layer are derived from the EGL. **(H-K) **Sagittal sections through E18 (H), P0 (I), P8 (J) and P20 (K) cerebella immunolabeled for calbindin (CaBP, green) and Zac1 (red), focusing on lobule IX. **(L) **A transverse section cut through the posterior cerebellum immunoperoxidase-stained at E18 with anti-Zac1. **(M) **A sagittal section through the cerebellum at E18 labeled with anti-Zac1 (red) and anti-BrdU (green; BrdU was injected into the pregnant dam 8 hours before the perfusion). Scale bars: 250 μm (G); 25 μm (H); 125 μm (L). EGL, external granular layer; GL, granular layer; IGL, internal granular layer; ML, molecular layer; PCL, Purkinje cell layer.

In cerebellar sections at E18 (Figure 1I), P0 (Figure 1G) and P3 (Figure 1J), we noted that Zac1 was expressed in several cell populations in addition to the medial nuclei. Firstly, at E18, Zac1 continued to be expressed in VZ progenitors, which are thought to give rise to late-born populations of GABAergic neurons in the cerebellar nuclei and cortex [37]. Zac1 immunoreactivity in the cerebellar VZ disappeared by around P3. Secondly, Zac1 expression was evident in the EGL of the posterior cerebellum beginning at E16 (presumptive lobules IX and X; Figure 2A). At this stage, no Zac1^+ ^cells were detected in the internal granular layer. By E18, Zac1^+ ^cells appeared to migrate inwards from the outer layer of the EGL into the immature molecular layer (Figures 1I and 2B,M). Zac1^+ ^cells were also observed in the granular layers of presumptive cerebellar lobules IX and X but not other lobules at P0 (Figure 2C), P3 (Figures 1J and 2D) and P8 (Figure 2E). Zac1 immunoreactivity in the granular layer started to disappear at around P12 (Figure 2F) and no cells in the granular layer expressing Zac1 were observed by P20 (Figure 2G) or in the adult (data not shown). Indeed, no Zac1 protein was detected in the adult mouse cerebellum (data not shown).

The pattern of anti-Zac1 immunostaining in the EGL of lobules IX/X and in adjacent internal layers suggested that Zac1^+ ^EGL cells may migrate to form a discrete population of neurons in the granular layer of these lobules. To follow this putative migration process, and to better localize Zac1^+ ^cells, we performed double immunolabeling with anti-Zac1 and anti-calbindin, a specific marker of all Purkinje cells in the adult cerebellum (for example, [38]) and subsets of Purkinje cells perinatally (for example, [29]). At E18, Zac1 and calbindin were expressed in distinct sets of cerebellar cells (Figure 2H). Zac1^+ ^cells were detected in the EGL, as anticipated, and were also interspersed throughout the calbindin^+ ^Purkinje cell layer, and in the underlying internal granule layer (Figure 2H). By P0, Zac1 expression was absent from the external granule layer, with most Zac1 immunoreactivity located beneath the Purkinje cell layer in the underlying granule layer (Figure 2I). At P8, Zac1 continued to be expressed in the internal granule layer, and was also detected in a subset of calbindin^+ ^Purkinje cells (Figure 2J). By P20, Zac1 expression was extinguished in both the internal granule and Purkinje cell layers (Figure 2K).

Taken together, the spatially dynamic expression profile suggested that Zac1^+ ^cells may migrate inwards to the internal granular layer from the EGL. To address this possibility further, we focused on E18, a dynamic stage during which Zac1^+ ^cells appeared to be migrating from the overlying EGL (Figure 2L). To label EGL cells and their derivatives, time-staged pregnant females were exposed at E18 to BrdU, a synthetic thymidine analog incorporated into newly synthesized DNA in dividing cells. Embryos were harvested 8 hours later and double-immunolabeled with anti-Zac1 and anti-BrdU (Figure 2M). Many Zac1^+ ^cells in the EGL, where proliferating progenitors are located, and in the underlying cellular layers, where differentiated neurons migrate, incorporated BrdU. The Zac1^+^/BrdU^+ ^labeling pattern was thus consistent with the hypothesis that Zac1^+ ^progenitors in the EGL give rise to neurons that take up a final position in the cerebellar cortex. The identity of these putative EGL-derived neurons was further investigated (see below).

### Zac1 is expressed in a subpopulation of Purkinje cells

As noted above, from P8 Zac1 immunoreactivity was detected in a subset of Purkinje cells located in the posterior cerebellum (cerebellar lobules IV to X; Figure 2J). Inspection of the high power images indicated that Zac1 protein was restricted to the nuclei of Purkinje cells, as expected of a transcriptional regulator (Figure 2E,F,J). No Zac1^+ ^Purkinje cell axons or dendrites were seen. Although not all Purkinje cells express Zac1, there was no clear evidence of parasagittal stripes (such as are revealed by other markers of early postnatal Purkinje cell subsets - for example, NG [29], HSP25 [39], and so on; reviewed in [40]). By P13/14, Zac1 expression had extended to include the majority of Purkinje cells in all lobules of the vermis and most of the Purkinje cells in the hemispheres (data not shown). There were also reproducible differences in the levels of Zac1 immunoreactivity between Purkinje cells in the anterior lobules (weaker) and those in the caudal portion of the vermis (stronger). Purkinje cell Zac1 expression was transient and was no longer detected by P20 (Figure 2G).

### Zac1 is expressed in a population of late-born Golgi cells

To identify the Zac1^+ ^cells in the granular layer of the posterior lobules, a variety of immunocytochemical markers were employed to distinguish between specific populations of cerebellar neurons. The calcium binding protein calretinin and metabotropic glutamate receptor (mGluR)1α is a marker for a subset of unipolar brush cells in the cerebellum [41-43], while parvalbumin, also a calcium-binding protein, is expressed by basket and stellate cells (*inter alia*) [44]. NG is a calmodulin-binding protein found in Golgi interneurons in the adult cerebellum [45]. In the adult mouse, cerebellar cortex NG immunoreactivity is restricted to the somata and dendritic arbors of a subset of Golgi cells [45,46]. In addition, weak expression is seen during postnatal development in a small subset of Purkinje cells, restricted primarily to the nodular zone, which is easily distinguished from the Golgi cells [29].

**Figure 3:**
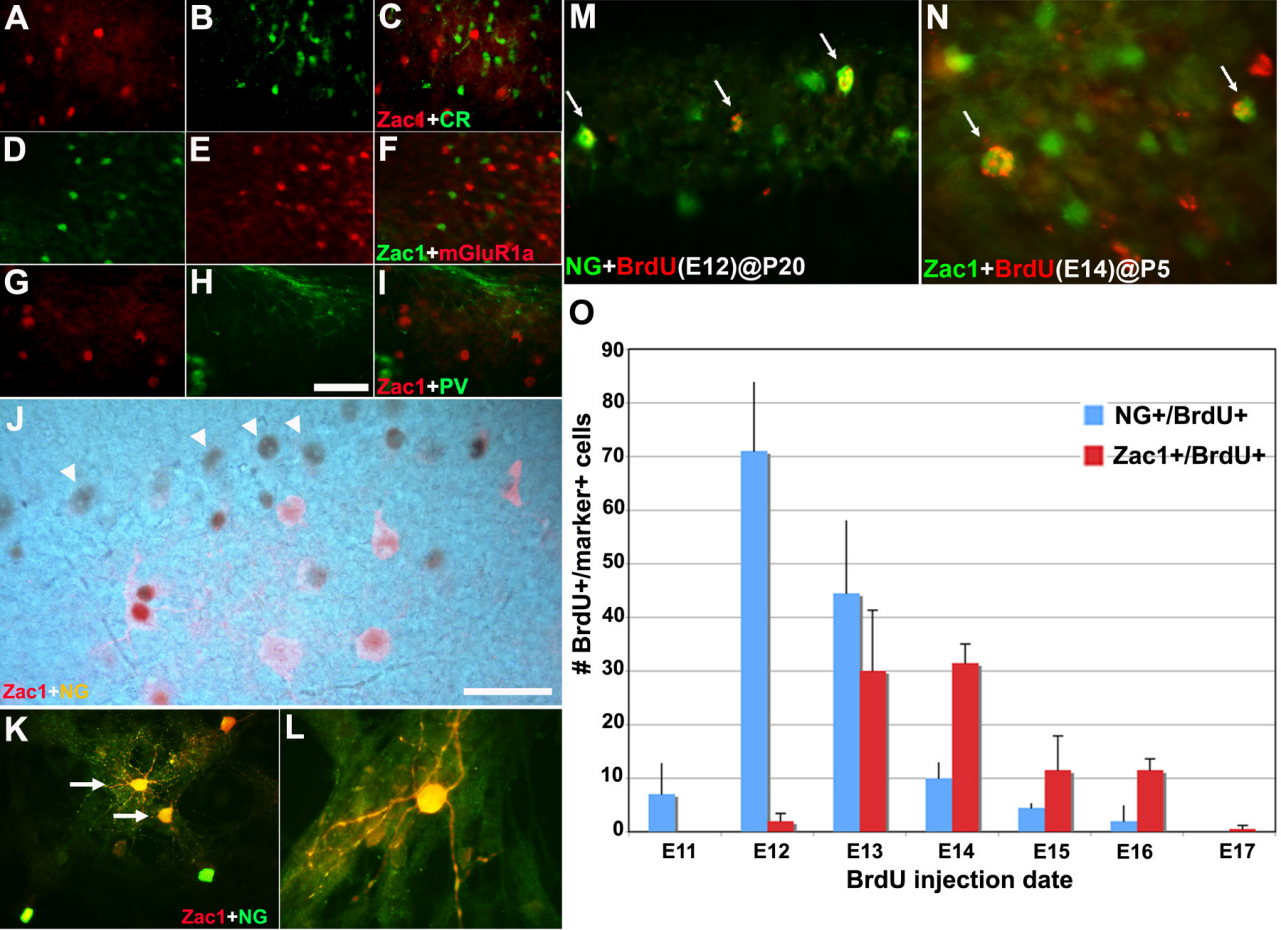
**Zac1 marks a subset of late-born cerebellar Golgi cells**. **(A-J) **Double immunofluorescence staining of P7 cerebella for Zac1 and calretinin (A-C), mGluR1α (D-F), parvalbumin (G-I) and NG (J; first immunoperoxidase stained with DAB for Zac1 (brown), then fluorescence stained for NG (red)). Arrowheads in (J) mark Purkinje cells. **(K,L) **Primary cultures of E18 mouse cerebella (21 days *in vitro*) co-labeled with anti-Zac1 (red) and anti-NG (green). Arrows in (K) mark Zac1^+ ^Golgi cells that co-express NG. **(M,N) **Birthdating of NG^+ ^(M) and Zac1^+ ^(N) Golgi cells, showing cells labeled with BrdU injections at E12 and analyzed at P20 (M) and BrdU injections at E14 and analyzed at P5 (N). **(O) **Quantification of the distribution of BrdU-immunostained Golgi cell nuclei in the P5 (for Zac1) and P20 (for NG) cerebellar cortex after exposure to BrdU at different stages during embryonic development (each stage, N = 4 pups, counted from sagittal sections; error bars represent standard deviation). Scale bars: 25 μm (H,J).

In P7 cerebella, calretinin^+ ^(Figure 3A-C), mGluR1α^+ ^(Figure 3D-F), and parvalbumin^+ ^cells (Figure 3G-I) were not co-labeled with Zac1^+ ^cells, suggesting that Zac1 is not expressed in subsets of unipolar brush, basket or stellate cells. In contrast, Zac1 and NG were co-expressed in a subset of cells in P7 cerebella (Figure 3J) and in E18 cerebellar cells dissociated and cultured for 21 days *in vitro *(Figure 3K,L), indicating that Zac1 is expressed in some NG^+ ^Golgi cells both *in vivo *and *in vitro*. Notably, the Zac1^+ ^cells that did not co-express NG may include: Golgi cells that have not yet initiated NG expression (or in which NG expression has been extinguished); a subpopulation of NG-negative Golgi cells, which have been previously described [46]; and/or other cerebellar cell types (for example, granule cells). While lineage tracing and additional marker analyses would be required to make these distinctions, we can conclude from our studies that Zac1 is expressed in a subset of NG^+ ^Golgi cells.

While the origin and birthdate of Golgi cells has been controversial, Zac1 is expressed in both of the putative progenitor zones, including the EGL [47-49] and the VZ of the fourth ventricle [47-49]. Based on previous studies [50-52], and our demonstration that Zac1^+ ^progenitors from the EGL migrate into the internal granular layer, where some cells continue to express Zac1 and differentiate into NG^+ ^Golgi cells, it may be that while most Golgi cells are derived from the neuronal epithelium of the fourth ventricle, a subset of Zac1^+ ^Golgi cells may also originate in the EGL. To better understand the origin and identity of Zac1^+ ^cells in the cerebellar cortex, we analyzed their birthdates, comparing them to the birthdates of NG^+ ^Golgi cells.

To analyze neuronal birthdates, the cerebellar cortex was sectioned at P5 (for Zac1 expression) or P20 (for NG expression) after a single exposure to BrdU at times between E11 and E17 (most Golgi cells are born prenatally [51-56]). Cells that were co-labeled with Zac1/BrdU and NG/BrdU were quantified in the posterior cerebellar lobules. The earliest Zac1^+ ^cells were born at E12, the numbers peaking at E13 to E14 and declining by E15 to E17 (Figure 3H). Based on the morphology and location of the Zac1^+^/BrdU^+ ^cells, and our demonstration that Zac1 is co-expressed with NG, we concluded that a subset of the birthdated Zac1^+ ^cells were Golgi cells. Consistent with this interpretation, there was some overlap in the period of NG^+ ^Golgi cell genesis. However, the bulk of the NG^+ ^Golgi cell population was born at E11, one day earlier than the Zac1^+ ^cells (Figure 3O). The NG^+ ^population also showed a protracted period of genesis, peaking at E12 and E13, with fewer born between E14 and E16 (Figure 3O). There are at least two possible interpretations of these data. The model we favor is that there are two distinct waves of cerebellar Golgi cell differentiation: an early wave including the generation of NG^+ ^cells that are born in the highest numbers between E12 and E13; and a second wave including the generation of Zac1^+ ^Golgi cells that reaches its peak between E13 and E14. As a corollary to this model, the Golgi cells that co-express Zac1 and NG may differentiate in an overlapping period. However, we cannot rule out a second possibility, which is that the Zac1^+ ^cells that are born outside of the window of NG^+ ^cell genesis are not Golgi cells. Nevertheless, with our studies, we have identified the temporal window during which Zac1^+ ^cells in the cerebellar cortex differentiate, and we have demonstrated that there is some overlap with the genesis of NG^+ ^Golgi cells, as expected.

### Zac1 expression is lost in the *Atoh1 *mutant EGL and Golgi cells

**Figure 4:**
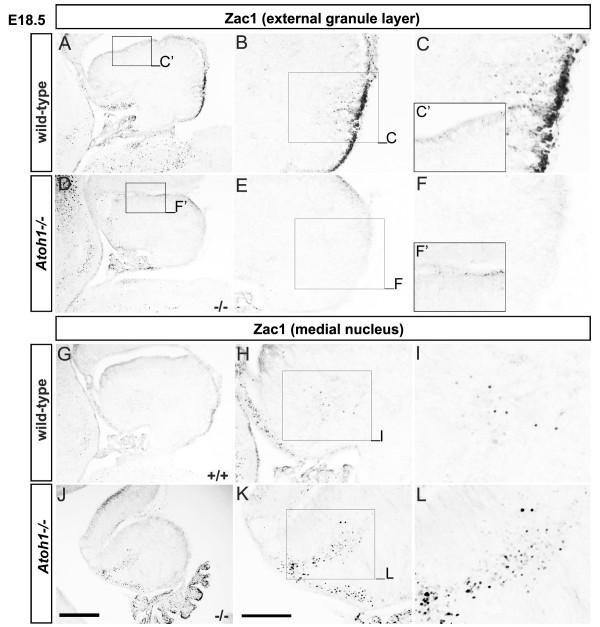
**Zac1**^**+ **^**Golgi cells are derived from the *Atoh1*/*Math1*-dependent EGL**. **(A-L) **Zac1 expression in E18 wild-type (A-C,G-I) and *Atoh1 *null (D-F,J-L) cerebellum. The rectangles in (A,B,D,E,H,K) indicate the locations of the higher magnification images in the adjacent panels. Scale bars: J = 250 μm (A,D,G,J); K = 125 μm (B,E,H,K).

To provide further proof that Zac1^+ ^Golgi cells are indeed derived from the EGL, we investigated Zac1 expression in mice homozygous for a null allele of mouse *Atoh1 *(*atonal homolog 1*; *Math1*). *Atoh1 *encodes a basic helix-loop-helix transcription factor that is expressed in the rhombic lip and its progeny, including EGL progenitors that differentiate into granule and Golgi cells, and glutamatergic projection neurons in the cerebellar nuclei [57,58]. Notably, *Atoh1 *is required to form the EGL and its derivatives [58], allowing us to determine if Zac1^+ ^cells in the posterior cerebellum, which includes NG^+ ^Golgi cells, are generated in the absence of the EGL. As observed previously, in the wild-type cerebellum at E18.5 (Figures 1I and 2B,L,M), Zac1 is strongly expressed in the posterior EGL and in inwardly migrating cells, some of which are NG^+ ^Golgi cells (Figure 4A-C). However, in E18.5 *Atoh1 *null mutants, Zac1^+ ^EGL cells and cells in the underlying cerebellar cortex, which includes Golgi cells, were sharply reduced in number (Figure 4D-F). This is consistent with the hypothesis that Zac1^+ ^cells in the cerebellar cortex, including a subset of Golgi cells, derive from the EGL.

We next asked if Zac1 expression was maintained in the medial cerebellar nuclei in *Atoh1 *mutants. We reasoned that because *Atoh1 *is not expressed in the cerebellar VZ [57], from which we propose Zac1^+ ^GABAergic neurons in the medial cerebellar nuclei arise, the maintenance of Zac1 expression in *Atoh1 *mutants would support our proposed lineage relationship. Notably, the cerebellar VZ gives rise not only to GABAergic neurons in the cerebellar nuclei, but also to other GABAergic populations, including Purkinje cells. Consistent with the lack of *Atoh1 *expression in this progenitor compartment, no difference was found in the number of Purkinje cells that differentiate in *Atoh1 *mutants, although a subset of Purkinje cells (approximately 28%) migrate aberrantly and form ectopias in the absence of *Atoh1 *function, revealing a role for the EGL and its derivatives (which do not form in *Atoh1 *mutants) in guiding Purkinje cell migration [59]. However, we have previously shown that in *scrambler *mutants, where Purkinje cell migration defects are even more severe, the organization of the deep cerebellar nuclei is intact [33]. We therefore speculated that the cerebellar nuclei would be intact in *Atoh1 *mutants, and proceeded with our analysis of Zac1 expression. As expected, Zac1^+ ^cells were detected in the medial nuclei in both wild-type (Figure 4G-I) and *Atoh1 *mutants (Figure 4J-L). This is consistent with our interpretation that Zac1^+ ^cells in the medial cerebellar nuclei are derived from the cerebellar VZ, and do not arise from the rhombic lip or its derivatives.

### Defects in the development of the medial cerebellar nuclei in Zac1 mutants

We found that Zac1 is expressed in a very restricted manner in the developing cerebellum, notably subsets of cerebellar progenitors in the VZ and EGL, and differentiated neurons in the medial nuclei and the posterior cerebellar cortex (Purkinje and Golgi cells). Such a restricted expression pattern suggested that *Zac1 *might play an essential role in the genesis of these cell types. To examine this possibility, we analyzed the *Zac1 *null cerebellum [15]. *Zac1 *is a maternally imprinted gene [11,12,25]. We therefore used *Zac1*^+m/- ^heterozygous mice, which had a wild-type maternal allele (that is, inactive due to imprinting), as the equivalent of null mutants. Notably, we previously verified that *Zac1*^+m/- ^animals are indeed null mutants and do not express Zac1 [23].

We first questioned whether the medial nucleus developed normally in the absence of *Zac1 *function. Approximately 80% of *Zac1*^+m/- ^animals die within the first few postnatal days for reasons currently unknown [15]. We therefore first analyzed development of the medial nucleus in E18.5 *Zac1*^+m/- ^embryos, hereafter referred to as *Zac1 *mutants. In both wild-type and *Zac1 *mutant embryos, Tbr1+ neurons were detected in the nuclear transitory zone, an expected result given that these neurons are derived from rhombic lip progenitors, which do not express Zac1 (Additional file 1A,B). We also examined the expression of *GAD1 *(Additional file 1C,D), which labels GABAergic interneurons in the developing deep cerebellar nuclei (DCN), including Zac1^+ ^cells (Figure 1K). However, because GAD1 also labels multiple cell populations in the cerebellar cortex, it was not possible to make definitive conclusions as to whether Zac1 was required for the formation of GABAergic neurons in the medial nucleus (Additional file 1C,D).

**Figure 5:**
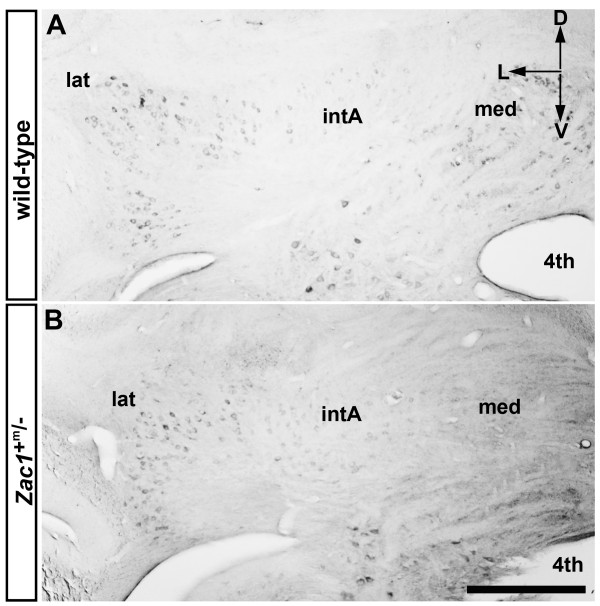
**A subdomain of the medial nuclei is missing in *Zac1 *null mice**. **(A,B) **A transverse section through the cerebellum of KLC3 expression in adult wild-type (A) and *Zac1 *mutant (B) animals. Strong and specific KLC3 expression is observed in all major cerebellar subnuclei, and in the vestibular complex (A). In the *Zac1 *null mouse cerebellar cortex, the anterior part of the medial nuclei is absent (B). D, dorsal; L, lateral; V, ventral; 4^th^, fourth ventricle; med, medial nucleus; intA, anterior interposed nucleus; lat, lateral nucleus. Scale bar: 250 μm.

Although most *Zac1*^+m/- ^pups die soon after birth, we were able to isolate four adult *Zac1*^+m/- ^animals for our analyses. To specifically label the cerebellar nuclei, we used the marker KLC3 [33]. In wild-type cerebella, strong and specific KLC3 expression was observed in all major deep cerebellar nuclei, including the lateral, interposed and medial nuclei, and in the vestibular complex (Figure 5A). KLC3 immunostaining suggests that the anterior part of the medial nuclei is missing in *Zac1 *mutant mice (Figure 5B); this corresponds to the loss of the normal *Zac1 *expression domain (equivalent to the M6 domain of Chung *et al. *[2]). The phenotype is restricted to the anterior medial nuclei; the interposed and lateral nuclei appear normal in *Zac1 *mutants (Figure 5B).

### Fewer Golgi cells are generated in Zac1 mutants

**Figure 6:**
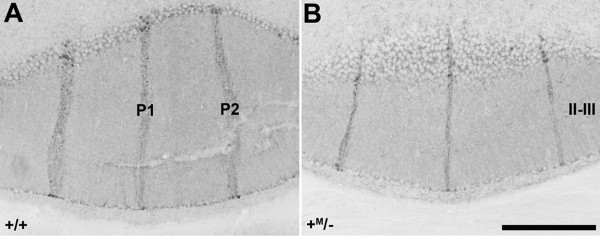
**Purkinje cell patterning is not disrupted in *Zac1 *null mice**. **(A,B) **Expression of zebrin II in the wild-type (A) and *Zac1 *null (B) adult cerebellum. Three characteristic narrow zebrin II stripes (P1+ at the midline and P2+ laterally to either side: for stripe terminology, see [60]) are observed in the anterior part (lobule II-III) of the wild-type cerebellum (A). The characteristic zebrin II-immunopositive stripes appear normal in the *Zac1 *null cerebellum (B). Scale bar: 250 μm.

We next investigated whether *Zac1 *mutants displayed defects in the generation of Purkinje and Golgi cells in the cerebellar cortex. To analyze Purkinje cells in adult *Zac1 *mutant mice, we first analyzed the expression of calbindin. No abnormalities in calbindin expression were detected in *Zac1 *mutant cerebella, suggesting that Purkinje cell development occurred normally (data not shown). To address the development of the Purkinje cell layer, we also examined the expression of zebrin II, which labels a subset of Purkinje cells that form parasagittal stripes [31]. In the wild-type cerebellum, three characteristic narrow zebrin II stripes (P1+ at the midline and P2+ laterally to either side: for stripe terminology, see [60]) were observed in the anterior zone of lobule II-III (Figure 6A); the characteristic anterior zone zebrin II^+ ^stripes appeared normal in the *Zac1 *null cerebellum (Figure 6B). The same was true of cerebellar patterning in other regions (data not shown).

**Figure 7:**
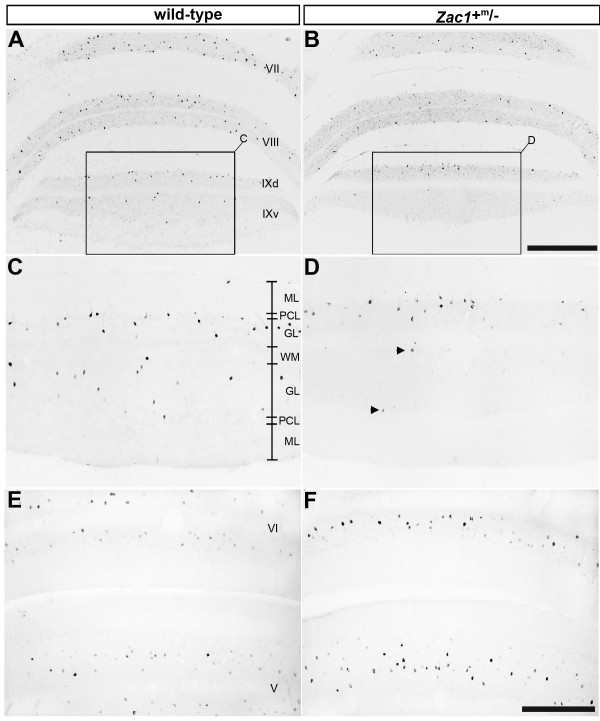
**Fewer Golgi cells are detected in *Zac1 *null mice**. **(A-F) **Expression of NG in transverse sections of wild-type (A,C,E) and *Zac1 *null (B,D,F) mice, focusing on lobules IX (C,D) and V/VI (E,F). Scale bars: B = 250 μm (A,B); F = 125 μm (C-F). GL, granular layer; ML, molecular layer; PCL, Purkinje cell layer; WM, white matter tract.

We next examined whether Golgi cells were generated normally in the absence of *Zac1 *function by using NG as a cell-type-specific marker. In cerebellar lobule IX of *Zac1 *null mutants (Figure 7B,D), the number of NG^+ ^Golgi cells was significantly reduced in *Zac1 *mutant mice compared to wild-type littermates (approximately 63% of wild-type levels: *Zac1 *mutant, 427 ± 11.53 NG^+ ^cells; wild type, 674 ± 10.57 NG^+ ^cells; *P*-value 0.0005; +/+, N = 4; +^m^/-, N = 3; Figure 6A,C). As predicted from the normal restriction of Zac1 expression to Golgi cells of the posterior vermis, these losses were restricted to cerebellar lobule IX. In contrast, counts of NG^+ ^Golgi cells in lobules V and VI showed no difference between *Zac1 *null and wild-type littermates (*Zac1 *mutant, 376 ± 7.69 NG^+ ^cells; wild type, 339 ± 13.05 NG^+ ^cells; *P*-value: 0.0131; +/+, N = 4; +^m^/-, N = 3; Figure 7E,F). We thus conclude that *Zac1 *is required for the generation of a subset of Golgi cells in posterior cerebellar lobule IX.

## Discussion

It is well established that transcription factors are required to specify neural cell identities and initiate subtype-specific neuronal differentiation programs throughout the central and peripheral nervous systems. Yet we are still a long way from having a comprehensive understanding of how neuronal diversity is generated during development, in particular in brain regions with complex morphologies and heterogeneous neuronal populations. The present data reveal a critical role for the transcription factor *Zac1 *in the generation of two specific cerebellar neuronal populations - one a GABAergic neuronal subset in the medial cerebellar nuclei, the other a subset of Golgi interneurons in the cerebellar cortex.

### *Zac1 *is required in the medial cerebellar nuclei

Cerebellar nuclear neurons can be broadly subdivided into small GABAergic interneurons and large glutamatergic projection neurons [61-63]. Early studies suggested that both neurochemically distinct subpopulations were derived from the neural epithelium lining the fourth ventricle (that is, the cerebellar VZ) [64-66]. However, recent expression and cell lineage tracing studies have instead indicated that Tbr1^+ ^glutamatergic projection neurons in the deep cerebellar nuclear neurons originate from Atoh1^+ ^progenitors in the rhombic lip [36,57,58]. A new model has therefore been proposed whereby the rhombic lip is the source for large diameter projection neurons in the cerebellar nuclei, while Ptf1a^+ ^and Pax2^+ ^progenitors in the cerebellar VZ give rise to GABAergic neurons, including small diameter neurons in the cerebellar nuclei [58,67,68]. The data presented here are consistent with this hypothesis. We found that Zac1 is also expressed in the cerebellar VZ, where Ptf1a and Pax2 are expressed. Furthermore, we demonstrate that Zac1 is expressed in a small subset of GABAergic interneurons in the medial nucleus, forming clusters that lie just ventral to the Tbr1^+^, glutamatergic projection neurons. Our data thus support the idea that Tbr1^+ ^and Zac1^+ ^nuclear neuron subsets are non-overlapping, and that these two populations of neurons have distinct embryological origins. KLC3 is a general marker to assess defects in the medial cerebellar nuclei as it is a specific marker of this region, which includes GABAergic interneurons [33]. The loss of KLC3 expression in the medial cerebellar nuclei in *Zac1 *null mice further supports a role for Zac1 in the formation of this cell population.

### *Zac1 *is required for the formation of a subset of cerebellar Golgi cells

Golgi cells [69] are large interneurons with their cell bodies dispersed throughout the granular layer and numerous radiating dendrites [70]. Golgi cell apical dendrites ramify through the molecular layer and are contacted by the axons of granule cells. In addition, a few basolateral dendrites extend in the granular layer (reviewed in [71]). The origin of Golgi cells has been controversial for decades. Originally Popoff [47] and Athias [48] suggested the EGL was the origin of these large neurons. This interpretation was supported by the more recent studies of Hausmann *et al. *[49], who used cerebellar transplantation to provide experimental evidence that Golgi cells originate from the EGL. However, in contrast, Ramon y Cajal [50] and Altman and Bayer [51] both concluded that Golgi cells are derived from the ventricular neuroepithelium. The same conclusion was reached by Zhang and Goldman [52] based on retroviral lineage tracing data. In fact, the cerebellar VZ is thought to give rise to all cerebellar GABAergic neurons, including Purkinje cells and interneurons such as Golgi, basket and stellate cells in the cerebellar cortex and small diameter interneurons in the cerebellar nuclei [58,68]. The data presented here reconcile both hypotheses. We identified a small subset of Zac1-immunopositive Golgi cells that are restricted to the posterior zone, and we provide birthdating evidence to suggest that these Golgi cells are derived from the EGL (presumably the population identified by Haussman *et al. *[49]). Notably, there is a precedent for the posterior EGL having a distinct molecular signature, as it is only in lobules IX and X that Lmx1a^+ ^precursors reside [72]. It is therefore interesting to speculate that the Zac1^+ ^Golgi cells could similarly originate from Lmx1a^+ ^progenitors in the posterior EGL. In contrast, we suggest that most Zac1-immunonegative Golgi cells instead originate from the neuroepithelium of the fourth ventricle.

Multiple Golgi cell subtypes have been identified based upon expression data but these are not reported to be regionally restricted (for example, [46,73,74]); unfortunately, there is no temporal overlap of the expression patterns of Zac1 and known subtype markers. The birthdating data show different, but overlapping, birthdate time windows for NG^+ ^and Zac1^+ ^cells. Immunohistochemical analysis with BrdU shows that most NG^+ ^Golgi cells are born between E12 and E14 whereas the majority of the Zac1^+ ^cells are born between E13 and E16. Thus, we speculate that most NG^+ ^Golgi cells derive from the neuroepithelium of the fourth ventricle between E12 and E14 (the EGL is not yet formed at this stage), while the smaller Zac1^+ ^subset of Golgi cells originate from the EGL at a later stage (E13 to E16). However, a *Zac1 *lineage trace using a *Zac1::cre *transgene and a reporter line (which presumably would label cells derived from the cerebellar VZ and not the rhombic lip) will be required to provide definitive proof for this interpretation.

Previous studies have suggested that *Zac1 *may play an important role in the differentiation of GABAergic progenitor cells. Valente and Auladell [75] showed that Zac1^+ ^neural progenitor cells in the third and fourth ventricles are GABAergic interneurons that leave the VZ and migrate to their final destinations. In this study, we have shown that the Zac1^+ ^medial nuclear neurons are GABAergic and will presumably become a part of the interneurons of the cerebellar nuclei, whereas the Tbr1^+ ^medial nuclear neurons will form later and become a subset of the cerebellar glutamatergic projection neurons. Golgi cells that express NG are GABAergic, whereas the Golgi cells devoid of NG are either GABAergic/glycinergic or glycinergic only [46]. There is also a small subset of Golgi cells (5 to 10%) that only express mGluR2 [46]. It is not clear why other GABAergic populations in the cerebellum do not require *Zac1 *function; from the fourth ventricle, these include Purkinje cells [76] and likely other inhibitory interneurons (for example, [37,77]).

It is not clear how *Zac1 *might act in the cerebellum. Clearly, the null allele lacks specific classes of neuron - subsets of Golgi cells and neurons of the medial nuclei. *Zac1 *has been best characterized as a tumor suppressor gene (*Zac1 *expression in human ovarian carcinoma cell lines was significantly decreased - 36% of them show an undetectable level of expression [16]). *Zac1 *expression was also reduced in primary breast cancer [78]. Consistent with this, Kamikihara *et al. *[79] showed a significant *Zac1 *mRNA reduction in primary ovarian cancer. However, other studies show that *Zac1 *controls normal development as a positive regulator (for example, [80]). Similarly, Ma *et al. *[23] recently proposed that *Zac1 *promotes the proliferation of neuronal progenitors in *Xenopus *retina. Thus, a likely explanation of the cerebellar phenotype is that *Zac1 *expression is required for the proliferation of the progenitors that give rise to two neuronal subclasses in the cerebellum.

## Conclusions

In this study, we used immunocytochemistry to analyze the Zac1 expression profile during embryonic and postnatal development in the murine cerebellar cortex. The results pointed to a key role for *Zac1 *in the formation of two specific subsets of GABAergic neurons: one arising from the fourth ventricle and forming a specific subgroup of the medial cerebellar nuclei, and a second derived from the EGL, which forms a specific subset of Golgi cells. This hypothesis was confirmed by analysis of the cerebellum in *Zac1 *null mice: specific defects were identified in both the medial nuclei and Golgi cells of the posterior vermis.

## Abbreviations

BrdU: 5-bromo-2-deoxyuridine; DCN: deep cerebellar nuclei; E: embryonic day; EGL: external granular layer; GABA: gamma-aminobutyric acid; HBSS: Hank's balanced salt solution; KLC: kinesin light chain; mGluR: metabotropic glutamate receptor; NG: neurogranin; P: postnatal day; PBS: phosphate buffered saline; VZ: ventricular zone.

## Competing interests

The authors declare that they have no competing interests.

## Authors' contributions

SC designed and carried out most of the experiments and drafted the initial manuscript. HM conducted the primary culture experiments. KA and KM generated and provided the *Math1 *null mice. CS provided the Zac1 antibody and *Zac1 *null mice and helped to revise the manuscript. RD carried out the E18.5 *Zac1 *mutant analyses. RH supervised the whole study, was responsible for its coordination, and edited the manuscript. All authors read and approved the final manuscript.

## Supplementary Material

Additional file 1 **Figure S1. Expression of *Tbr1 *and *GAD1 *in medial cerebellar nuclei in *Zac1 *mutants**. **(A,B) **Expression of *Tbr1 *in E18.5 wild-type (A) and *Zac1*^+m/- ^(B) deep cerebellar nuclei. **(C,D) **Expression of *GAD1 *in E18.5 wild-type (C) and *Zac1*^+m/- ^(D) deep cerebellar nuclei. Med, Medial cerebellar nuclei. Click here for file
