# Supplementary figures and images for: Zac1 plays a key role in the development of specific neuronal subsets in the mouse cerebellum
Source: Neural Dev. 2011 May 18;6:25. doi: 10.1186/1749-8104-6-25 (PMC3113315; doi:10.1186/1749-8104-6-25)

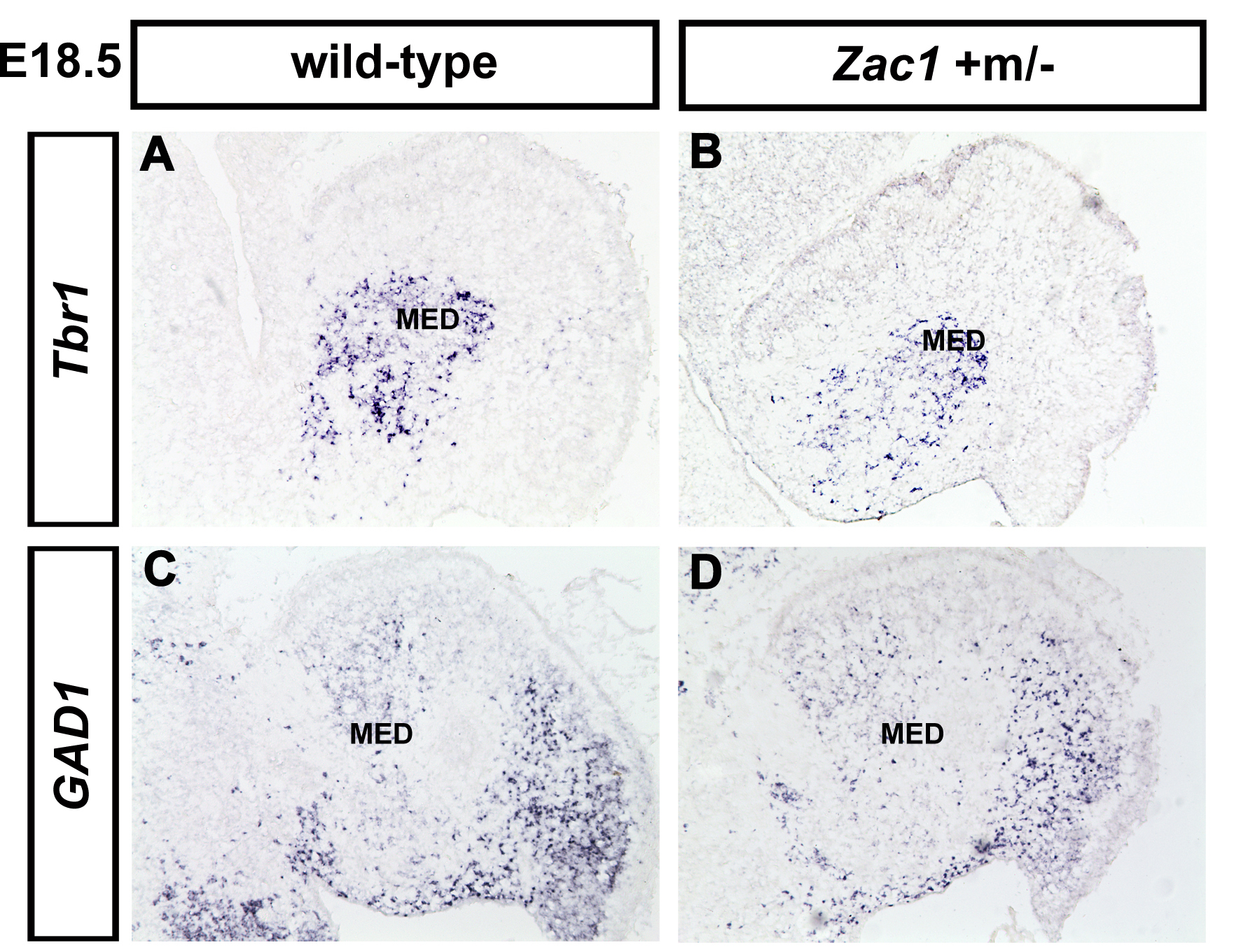

Supplement: Additional file 1 — Figure S1. Expression of Tbr1 and GAD1 in medial cerebellar nuclei in Zac1 mutants. (A,B) Expression of Tbr1 in E18.5 wild-type (A) and Zac1+m/- (B) deep cerebellar nuclei. (C,D) Expression of GAD1 in E18.5 wild-type (C) and Zac1+m/- (D) deep cerebellar nuclei. Med, Medial cerebellar nuclei. [file 1749-8104-6-25-S1.JPEG]
